# Tracking reduction of water lead levels in two homes during the Flint Federal Emergency

**DOI:** 10.1016/j.wroa.2020.100047

**Published:** 2020-03-03

**Authors:** Anurag Mantha, Min Tang, Kelsey J. Pieper, Jeffrey L. Parks, Marc A. Edwards

**Affiliations:** Virginia Tech, Charles E. Via, Jr. Department of Civil and Environmental Engineering, 1145 Perry St., 418 Durham Hall, Blacksburg, VA, 24061, United States

**Keywords:** Sequential sampling, Profiling, Lead in water, Corrosion control, Premise plumbing

## Abstract

A Federal Emergency was declared in Flint, MI, on January 16, 2016, 18-months after a switch to Flint River source water without phosphate corrosion control. Remedial actions to resolve the corresponding lead in water crisis included reconnection to the original Lake Huron source water with orthophosphate, implementing enhanced corrosion control by dosing extra orthophosphate, a “Flush for Flint” program to help clean out loose leaded sediment from service lines and premise plumbing, and eventually lead service line replacement. Independent sampling over a period of 37 months (January 2016–February 2019) was conducted by the United States Environmental Protection Agency and Virginia Tech to evaluate possible human exposure via normal flow (∼2–3 L/min) sampling at the cold kitchen tap, and to examine the status of loose deposits from the service line and the premise plumbing via high-velocity flushing (∼12–13 L/min) from the hose bib. The sampling results indicated that high lead in water persisted for more than a year in two Flint homes due to a large reservoir of lead deposits. The effects of a large reservoir of loose lead deposits persisted until the lead service line was completely removed in these two anomalous homes. As water conservation efforts are implemented in many areas of the country, problems with mobile lead reservoirs in service lines are likely to pose a human health risk.

## Highlights

• Lead levels took 3 years to recover in two homes after corrosion control disruption.
• Low water use due to fear of water safety may have prolonged the recovery time.
• A large reservoir of loose leaded deposits persisted until LSL was replaced.
• Normal flow rate sampling can simulate human exposure.
• High flow rate sampling can quantify status of mobile deposits.

## Graphical abstract

**Figure:**
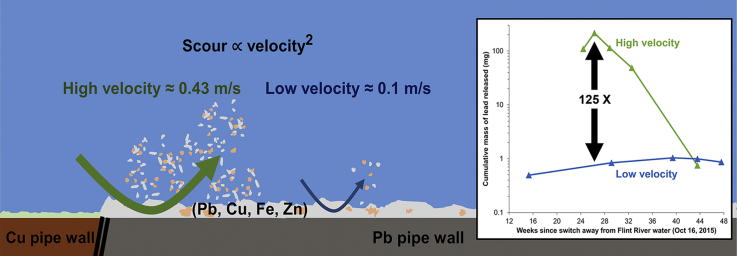

## 1 Introduction

After decades of steady decline in mean blood lead (Pb) levels in the United States (Dignam et al., 2019), the recent Flint Federal Emergency has increased concern of exposure from Pb in drinking water (Davis et al., 2016). The Natural Resources Defense Council (NRDC) reported that more than 5300 public water systems in the United States, serving over 18 million people, were in violation of the Environmental Protection Agency (EPA) Lead and Copper Rule (LCR) in 2015 due to failures in water Pb monitoring, corrosion control, and reporting to the public and regulators (EPA, 1991; Olson and Fedinick, 2016).

### 1.1 Source and occurrence of lead in water

Lead in water is derived from corrosion of lead service lines (LSLs), Pb goosenecks, Pb-lined steel pipes, Pb solder, galvanized iron pipes, and leaded brass fittings. All of these sources of Pb can act as reservoirs for Pb release and corrosion control treatment (CCT) is implemented at water utilities to optimize water chemistry to minimize Pb release (Clark et al., 2015; EPA, 2016b, 1991; HDR Engineering Inc., 2009; Lytle and Schock, 1996; Subramanian et al., 1995; Tang et al., 2018; Triantafyllidou and Edwards, 2012). Popular CCT methods include orthophosphate corrosion inhibitors and pH/alkalinity adjustments and the revised LCR re-emphasizes the importance of these approaches (Clark et al., 2014; EPA, 2016b; EPA, 2019).

There is evidence that water Pb release may now be increasing in some cities due to higher temperatures, water conservation, use of chloramine, road salt use and other factors (Roy et al., 2018). Changes in water chemistry can also exacerbate Pb release by destabilizing scales/rust on pipe that formed over decades or centuries, mobilizing Pb from service lines and/or in-home plumbing (Clark et al., 2015; Edwards et al., 2009; Lytle et al., 2019; Masters and Edwards, 2015; McFadden et al., 2011; Pieper et al., 2017; Sandvig et al., 2008; Tang et al., 2018; Triantafyllidou and Edwards, 2012), or through Pb “seeding” onto galvanized steel pipes and direct Pb release from zinc coating on galvanized steel pipes (HDR Engineering Inc., 2009; Clark et al., 2015). In addition, the presence of copper upstream of an LSL has been known to create serious problems with sustained particulate Pb release due to deposition corrosion (Britton and Richards, 1981; Hu et al., 2012; St. Clair et al., 2012).

The mobilization of Pb in water will vary as a function of plumbing configuration, Pb materials in use, sampling locations, water use patterns, water temperature, and flow rates during sampling (Britton and Richards, 1981; Cartier et al., 2012b, 2012a; Clark et al., 2014; Del Toral et al., 2013; Edwards and Dudi, 2004; Pieper et al., 2015; Sandvig et al., 2008; St. Clair et al., 2016; Tang et al., 2018). For example, flushed samples collected at higher water velocity tend to mobilize higher levels of loose particulate Pb (Cartier et al., 2012a; Clark et al., 2014; Masters et al., 2016; Pieper et al., 2017; Triantafyllidou and Edwards, 2012). In one well-controlled lab study, the mean Pb level in water flushed from Pb-copper partial service line testing rigs at high flow of 10 L/min was 10,136 μg/L – 216 times higher than the Pb levels (47 μg/L) obtained at low flow of 2 L/min (Masters et al., 2016). Another study based on field data during the Washington D.C. lead crisis showed hundreds of times higher particulate Pb in water at 10 L/min versus 0.4 L/min (Triantafyllidou and Edwards, 2012). Aerators on low-flow fixtures and showers reduced consumption of water due to contamination events, and/or voluntary conservation due to high water rates will reduce the velocity and volume of water flowing through homes (Nguyen et al., 2012). Low flows are problematic because water stays in contact with the pipes longer, increasing soluble Pb in water. Low flows can also increase particulate Pb release due to accelerated corrosion rates (Arnold and Edwards, 2012; Del Toral et al., 2013).

### 1.2 Remedial flushing to reduce lead in water

The notion of remedial flushing for removal of loose iron rust from water systems is well established (Benson et al., 2012) and can be extended to reducing Pb release in new buildings with water conservation features through automated flushers (Nguyen et al., 2012). Flushing pipes at high-velocity flow has also been shown to reduce Pb levels after LSL replacements (Brown and Cornwell, 2015; Cornwell et al., 2018; Deshommes et al., 2016). While in some cases, a one-time flush event is effective in remediating Pb levels in water (Elfland et al., 2010; Cornwell et al., 2018; Pieper et al., 2015), in other cases, water Pb problems may persist for a much longer duration due to changes in water chemistry (Lytle et al., 2019; Masten et al., 2016).

### 1.3 Fingerprinting metal correlations to identify the source of lead

Identifying the source of water Pb can help interpret the nature of the corrosion problem, which in turn could help identify common plumbing characteristics of homes at risk within a city. Sequential sampling coupled with “fingerprinting” metal correlation techniques and detailed information about the plumbing has provided such diagnostic insights in the past (HDR Engineering Inc., 2009; Clark et al., 2015; Deshommes et al., 2010; Lytle et al., 2018; Pieper et al., 2017). Specifically, tin co-occurrence with Pb indicates solder, zinc and copper co-occurrence indicates brass, and cadmium co-occurrence indicates galvanized iron (Clark et al., 2015; Deshommes et al., 2010; Edwards and Triantafyllidou, 2007; Lytle et al., 2018; Pieper et al., 2017, 2015).

### 1.4 Flint case study

The City of Flint, Michigan failed to continue CCT when switching from Lake Huron water with orthophosphate to a Flint River source without orthophosphate in April 2014, creating a major water Pb contamination event (Davis et al., 2016; Edwards et al., 2015; Pieper et al., 2018; Masten et al., 2016; Schwake et al., 2016). After a local and Federal Emergency was declared, relief agency responses to mitigate the problems included reconnecting to the original Lake Huron source with orthophosphate (October 16, 2015), implementation of enhanced corrosion control with extra phosphate (December 09, 2015), and a “Flush for Flint” program in homes to clean Pb sediments from home plumbing systems (May 1–15, 2016) (EPA, 2016a; Pieper et al., 2018).

The Pb in water levels decreased from these remediation efforts. Four rounds of sampling in Flint by the EPA indicated an overall decrease in average particulate Pb levels from 37 μg/L in January–March 2016 to 9 μg/L in November 2016 at cold tap collected at normal flow (Lytle et al., 2019). Similarly, five rounds of sampling in Flint by Virginia Tech between August 2015 and August 2017, showed that the 90th percentile total Pb of first draw samples reduced from 26.8 μg/L to 7.9 μg/L (Pieper et al., 2018). Most of the Pb released after the reintroduction of corrosion control was likely particulate Pb, due to dislodging of pipe scale (Clark et al., 2014; Pieper et al, 2017, 2018). There were still a few homes where severe problems with high Pb persisted (Abokifa et al., 2020; Pieper et al., 2018), as was the case in the aftermath of another major water Pb contamination event in Washington D.C. 2001–2004 (Edwards and Dudi, 2004; Edwards, 2014; McFadden et al., 2011).

Two of the most problematic Flint homes (Lytle et al., 2019; Pieper et al., 2018) were intensively sampled by two independent teams including the EPA and Virginia Tech, with a total of 462 water samples collected at both homes (209 at home A and 253 at home B) over a period of 37 months (January 2016–February 2019). It was determined early on that during the system recovery the vast majority of the Pb was particulate and any soluble Pb was being precipitated by the high levels of phosphate being added during enhanced CCT (Fig. S4; Pieper et al., 2017). While the EPA sampling was conducted at normal flow in order to quantify recovery of Pb levels at the kitchen tap, Virginia Tech sampling was conducted to quantify the duration and magnitude of Pb release from reservoirs of loose particulates by flushing at high flow rates from the hose bib. We hypothesized that repeated flushing at the highest possible flow from a home hose bib would reduce Pb levels in water by progressively cleaning out reservoirs of loose Pb deposits in the pipes. We also hypothesized that water samples collected at normal flow (from the kitchen tap) would not always provide insights to this reservoir of loose Pb deposits, even though it is the best measure of human exposure and health risks.

The objectives of this study were to compile and compare the results of these two complementary datasets, which tracked the recovery of premise plumbing systems impacted by interruptions in CCT to (1) evaluate the relative effectiveness of high-velocity flushing as a way to clean out loose Pb deposits in premise plumbing, (2) identify the source of Pb in water using a “fingerprinting” metal co-occurrence approach, and (3) track recovery of water Pb levels after interventions of corrosion control, flushing and service line replacement.

## 2 Materials and methods

### 2.1 Sampling site selection and plumbing survey

Two homes (A and B) had LSLs and high levels of Pb in water during sampling by Virginia Tech in August 2015 and March 2016 (Fig. S1). Both Virginia Tech and EPA selected these two homes as part of their intensive federal emergency monitoring program after implementing enhanced CCT in Flint, MI (FTF, 2016; Lytle et al., 2019). The detailed home plumbing and service line materials are described and cumulative volumes in each plumbing section are calculated in Tables S1–S4. In brief, home A had both city- and private-owned LSLs connecting at the shut-off valve, with mostly Pb-bearing brass fittings, Pb solder joints, and copper piping within the home. Home B had a city-owned copper service line and private-owned LSL, with Pb-bearing brass fixtures, Pb solder joints, and copper piping within the home within the home. The entire LSL was replaced with a copper pipe in home A on February 01, 2017, and in home B on September 28, 2016.

### 2.2 Sequential sampling protocols

Virginia Tech conducted sequential sampling from the hose bib at homes A and B at a high flow rate of approximately 12-13 L/min to maximize Pb mobilization (Table 1). On April 04, 2016, after 4 months of enhanced CCT, sequential sampling with the hose bib sequential (HBS) protocol (Fig. S2) was used to collect up to 16 consecutive 1-Liter water samples from both homes after 6-h of stagnation to establish a baseline for Pb in water levels. To examine the impact of extended flushing at high flow rates on removal of loose Pb deposits, a hose bib longer duration sequential sampling protocol (HBLD) (Fig. S2) was devised to include 8 alternating 1-Liter water samples (1st, 3rd, 5th …. 15th liter), and then 6 1-Liter water samples with 5 min flushing between each. The HBLD protocol was used for both homes on April 17, 2016, and thereafter, to examine the Pb removal following the “Flush for Flint” program in May 2016.

**Table 1:** Sampling dates and protocols for home A and B.

Dates	Home A	Home B
Jan 31, 2016	KTS	–
Feb 08, 2016	–	KTS
Apr 04, 2016	HBS	HBS
Apr 17, 2016	HBLD	HBLD
May 05, 2016	HBLD	HBLD
May 07, 2016	KTS^a^	KTS^a^
May 31, 2016	HBLD	HBLD
Jul 18, 2016	KTS	KTS^a^
Aug 16, 2016	–	HBLD & KTS^b^
Sep 13, 2016	KTS	KTS^a^
Sep 28, 2016	–	LSL Replaced
Nov 01, 2016	–	HBLD
Nov 08, 2016	–	KTS^a^
Nov 26, 2016	KTS	–
Feb 14, 2017	LSL Replaced	–
Mar 09, 2017	HBLD & KTS^b^	HBLD & KTS^b^
Feb 21, 2019	HBS & KTS^b^	HBS & KTS^b^

KTS: kitchen tap sequential sampling. HBS: hose bib sequential sampling. HBLD: hose bib long duration sampling. LSL: lead service line.

a One or more 1-Liter samples were replaced by two 500 mL bottles to increase the resolution of Pb concentrations.

b KTS sampling conducted by Virginia Tech using EPA Protocol.

In contrast, the parallel EPA sequential sampling was from the kitchen faucet for both homes A and B, using a flow of approximately 2–3 L/min from the tap with the aerator on (Table 1). The kitchen tap sequential (KTS) (Fig. S2) samples included two 125 mL bottles followed by up to 16 1-Liter sequential water samples after 6-h of no water use. The EPA sampling flow scenario represents typical water use in a household (Cartier et al., 2012a; Clark et al., 2014; Del Toral et al., 2013). The first sampling was conducted on January 31, 2016, in home A and February 08, 2016 in home B. Thereafter were instances. when EPA used two 500 mL bottles, to replace one or more 1-Liter bottles within a sampling round, at the discretion of the sampling team.

### 2.3 Water sample analysis

All water samples collected by Virginia Tech were shipped to Blacksburg, VA within two business days of collection for total elemental analyses (e.g., Pb, Fe, Cu, P). Upon receipt, each water sample was acidified with 2% concentrated nitric acid by volume and held for a minimum of 16 h. Samples were analyzed for metals and phosphate by inductively coupled plasma mass spectrometry (ICP-MS; Thermo Scientific Thermo Electric X Series) based on Standard Method 3125B (APHA, AWWA, WEF, 1998). Samples of blanks and spikes of known concentrations were measured after every 10 samples for quality assurance and quality control (QA/QC). All water samples collected by the EPA were analyzed for total metals (e.g., Pb, Fe, Cu, Zn) using Method 200.7/200.8 after acidifying to 3% acid by volume and held for a minimum of 16 h (Lytle et al., 2019). Laboratory blanks and spikes were measured every 20 samples for QA/QC.

### 2.4 Pipe scale and brass scraping analysis

For LSL scale, a scraping was collected along the entire length of the pipe exhumed from the property. Then, 5 mg samples were taken in triplicate and dissolved in 20% nitric acid solution and digested at 60 °C for >16 h. For brass and solder, a single composite scale sample with a total weight of at least 5 mg was taken from each home and prepared for total metals analysis as previously outlined. If samples contained visible particulate after the 16 h period, 2% hydroxylamine was added and the sample was held for an additional 16 h at 50 °C (Masters and Edwards, 2015). Afterwards, the digested samples were analyzed by ICP-MS as outlined in section 2.3. Metal ratios (e.g., Pb:Fe, Pb:Cu) were calculated using elemental analyses.

### 2.5 Statistical analysis

All data were analyzed using R Studio (R version 3.5.3) and an alpha of 0.05 was used as a measure of significance. As Pb concentration data were non-normal (Shapiro-Wilk test, p < 0.05), Wilcoxon and Mann-Whitney U tests were used to compare Pb concentration among the different sampling rounds and sampling locations. Spearman’s rank correlation (ρ) was used to determine correlations between Pb, Fe, phosphate, and Zn in water. Relative standard deviation (RSD = standard deviation/mean) was used to evaluate the variability of Pb in water (Masters et al., 2016).

## 3 Results and discussion

### 3.1 Water lead levels during high-velocity flushing

Pb concentrations in water flushed from hose bibs and the associated variability for home A and B were monitored by Virginia Tech starting in April 2016 after 4 months of CCT, during and after the flushing program, and after LSL replacement.

#### 3.1.1 Trends in water Pb levels for home A

**Fig. 1:**
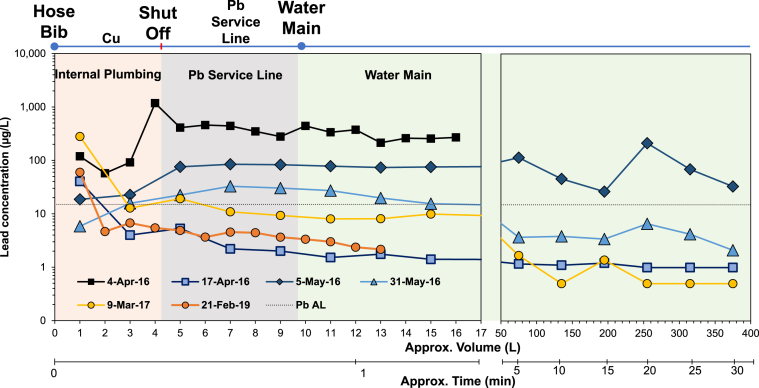
Lead concentrations in water as a function of water volume, flushed time, and plumbing layout for home A. Dashed line represents the EPA Pb action level (AL) of 15 μg/L. Samples below the minimum reporting level (1.0 μg/L) were reported at 0.5 μg/L.

The initial sampling at normal flow in August 2015 detected 406 μg/L, 9.7 μg/L, and 6.1 μg/L in the first, second, and third draw samples, respectively (Fig. S1). After 4 months of orthophosphate CCT, the Pb in water level collected on April 04, 2016, was 119.3 μg/L in the first draw, 8 times the EPA action level (AL) of 15 μg/L (Fig. 1). During the 16-Liters of flushing, Pb levels remained at relatively high levels of 57.3–1180.0 μg/L. After 10 L, all of the water that sat stagnant in the service line should have been replaced by fresh water from the main, and most of the Pb in the subsequent 11–16 L samples was suspected to reflect scouring of loose deposits from pipes.

When the flushing event was repeated two weeks later, on April 17, 2016, the median Pb in water was significantly reduced from 307.5 μg/L (April 04, 2016) to 1.5 μg/L (Mann-Whitney U test, p < 0.0001), illustrating the benefits of the one-time flushing event in this home. However, on May 5, 2016, the median Pb was again high relative to sampling on April 17, 2016, increasing to 74.4 μg/L (Wilcoxon Signed Rank Test, p < 0.0001).

Two weeks after the citywide “Flush for Flint” program, on May 31, 2016, the Pb in water significantly decreased to a median of 11.0 μg/L (Wilcoxon Signed Rank Test, p < 0.0001) and ranged 2.1–32.8 μg/L. The reduction in median Pb levels at home A potentially suggested that the city-wide flushing program helped with speeding up the recovery for home A’s infrastructure as predicted (EPA, 2016a). To eliminate the Pb sources in home A, the entire LSL was replaced with a Cu pipe on February 14, 2017. Three weeks after the LSL replacement, the overall Pb in water levels continued to decrease with a median of 8.1 μg/L (p < 0.0001), compared to levels in the previous May. Even though the median Pb levels decreased from April 2016 to March 2017 (p < 0.0001), on March 03, 2017, the first draw sample after LSL replacement still contained a concerning Pb level of 279.9 μg/L, which was 19 times the EPA AL of 15 μg/L.

In a follow-up sampling conducted on Feb 21, 2019, the first draw sample contained 59.9 μg/L, likely due to the brass hose bib as indicated by co-occurrence of elevated Zn and Cu with the Pb in water. All the remaining flushed samples were below 5 μg/L, suggesting the vast majority of loose Pb deposits had finally been removed. High Pb in water levels in the first sample collected from the hose bib was likely due to the infrequent use of the fixture (personal communication with homeowner) and is consistent with observations in other studies (Cartier et al., 2012a; Lytle and Schock, 1996; McFadden et al., 2011).

#### 3.1.2 Trends in water Pb levels for home B

The Pb in water for home B presented a different pattern than home A. The Pb in water levels measured on April 04, 2016, were 31.6 μg/L in the first draw, with the remaining 11 water samples containing 14.5-775.9 μg/L Pb. Another 2 weeks of enhanced CCT (April 17, 2016) did not significantly improve median Pb levels due to large variability (Mann-Whitney U Test, p = 0.065). The Pb in water during this prolonged flushing (up to 30-min) was also highly variable and ranged from 2.6-1308.0 μg/L*.*

The “Flush for Flint” program was likely not as successful in removing leaded deposits in this home, as the median Pb remained at high levels on May 5, 2016 (Wilcoxon Test, p = 0.18) and May 31, 2016 (p = 0.55). Compared to the median level at 20.2 μg/L on April 17, 2016, the median Pb in water increased to 50.6 μg/L on May 5, 2016, and further to 104.6 on May 31, 2016. In any case, water Pb levels did not recover at this home, perhaps due to the presence of Cu pipe upstream of the Pb LSL, a configuration which has been known for decades to sometimes create massive problems with Pb release (Britton and Richards, 1981; Hu et al., 2012; St. Clair et al., 2012). For instance, a 1981 study in Scotland noted that no amount of flushing from such configurations where Cu is present upstream of Pb could reduce Pb in water to allowable levels (Britton and Richards, 1981).

The entire Pb–Cu partial service line in home B was replaced with Cu pipe on September 28, 2016. On March 03, 2017, the median Pb level significantly decreased to 2.5 μg/L (Wilcoxon Test, p = 0.0002) versus May 31, 2016. During the follow-up sampling on February 21, 2019, the first draw sample contained 57.1 μg/L, which was likely due to the hose bib after an estimated 6 months stagnation, while all other samples collected were below 5 μg/L*.* As it has been well documented that an LSL is the most concentrated source of Pb in drinking water (Cartier et al., 2012a; Del Toral et al., 2013; Deshommes et al., 2010; Lytle et al., 2019, 2018; Masters et al., 2016; Olson et al., 2017; St. Clair et al., 2016), replacing the LSL and having enhanced CCT in water was effective for reducing Pb at home B.

#### 3.1.3 Variability in water Pb levels

High inherent variability of Pb in water levels after the interrupted corrosion control was observed for both homes as indicated by a 52–229% RSD for all sampling rounds (Table 3), which was 2–4 times higher than the range of 21–80% reported by Masters et al., (2016) for systems around the U.S. Three sampling rounds with relatively large RSDs of 122–283% were collected on April 17, 2016, May 05, 2016 and March 09, 2017, which demonstrates the semi-random and sporadic nature of Pb release to water due to disruption of pre-existing pipe scale (Cartier et al., 2011; Clark et al., 2014; Masters et al., 2016; Pieper et al., 2017).

#### 3.1.4 Effectiveness of high-velocity flushing

While a significant reduction in Pb levels was observed in both homes at the end of the monitoring period (April 2016–February 2019), it took repeated high-velocity flow events over many months to remove non-durable scale from the plumbing. After the disruption in CCT occurred in Flint in April 2014, significant water Pb problems persisted until the LSL was replaced in both homes, which was over one year after the switch back to Lake Huron source water, and over 10 months after the implementation of the enhanced corrosion control. One-time remedial flushing on April 04, 2016, only showed a short-term reduction in Pb levels in these two cases. Follow-up indicated that the homeowner at home A was elderly, was using bottled water for drinking, and had even begun taking short showers (less than 5 min) due to fears of water safety (personal communication, homeowner). We speculate that the relatively low water use rates (home A), and copper upstream of LSL configuration (home B) were contributing factors to the slow reduction in Pb levels in both homes.

### 3.2 Source of lead in water

#### 3.2.1 Lead sources from LSL, lead solder, and brass fittings during stagnation

**Fig. 2:**
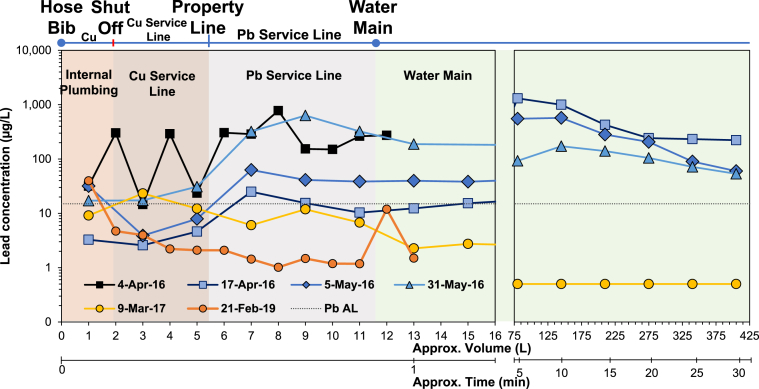
Lead concentrations in water as a function of water volume, flushed time, and plumbing layout for home B. Dashed line represents the EPA Pb action level (AL) of 15 μg/L. Samples below the minimum reporting level (1.0 μg/L) were reported at 0.5 μg/L.

Both homes had LSLs and internal copper pipes with leaded solder. The shutoff and gate valves, nipples, water meter, and tee joints were all lead-bearing brass. It was circumstantially determined that the LSL was the primary source of Pb in water levels, as Pb spikes in both homes coincided with water representative of that sitting stagnant in the LSL (Fig. 1, Fig. 2). The LSL scale scraping derived from both homes had 4-54 times higher Pb:Fe and 15–961 times higher Pb:Zn indicating predominantly leaded scale on the inside of the service lines (Table 2). The Pb:Zn ratio in water from both homes until May 31, 2016, was in the range of 2.1–100, which is much higher than the Pb:Zn ratio in brass of 0.06–4.41. Moreover, lead-tin solder was effectively ruled out as a source of elevated Pb levels due to the absence of Sn in the water with high Pb. After LSL replacement, brass fixtures became the major source of Pb in water in both homes, with a Pb:Zn ratio in this first draw water (0.68–1.37) close to the Pb:Zn ratio in the brass (0.09–0.96).

**Table 2:** Metal ratios from LSL scale, solder, brass and water at home A and B.

Metal Ratios	LSL Scale Scraping	Solder Scraping	Brass Scraping	Water Samples				
				Apr 04, 2016	Apr 17, 2016	May 05, 2016	May 31, 2016	Mar 09, 2017
	Home A							
Pb:Fe	53.8 ± 36.2	1.42	0.08	0.50 ± 0.14	0.08 ± 0.14	0.40 ± 0.13	0.17 ± 0.14	0.24 ± 0.24
Pb:Zn	961 ± 647	4.41	0.09	17.3 ± 11.6	0.28 ± 0.13	16.6 ± 21.1	2.10 ± 1.12	0.68 ± 0.42
Pb:Cu	43.7 ± 26.7	0.23	0.05	4.12 ± 2.40	0.43 ± 0.15	2.22 ± 1.25	1.27 ± 0.93	0.12 ± 0.1
Cu:Zn	21.3 ± 2.0	19.58	1.88	3.77 ± 1.18	0.63 ± 0.13	9.49 ± 14.1	1.71 ± 0.67	9.06 ± 7.86
**Metal Ratios**	**LSL Scale Scraping**	**Solder Scraping**	**Brass Scraping**	**Water Samples**				
				**Apr 04, 2016**	**Apr 17, 2016**	**May 05, 2016**	**May 31, 2016**	**Mar 09, 2017**
	**Home B**							

Pb:Fe	4.4 ± 0.6	0.13	52.55	0.56 ± 0.20	0.64 ± 0.54	0.77 ± 0.52	0.45 ± 0.26	0.03 ± 0.03
Pb:Zn	15.4 ± 1.0	0.06	0.96	85.5 ± 86.7	100 ± 164	56.7 ± 77.8	49.2 ± 41.9	1.37 ± 1.45
Pb:Cu	19.3 ± 6.85	0.03	0.05	4.34 ± 3.24	2.75 ± 2.90	3.32 ± 3.02	3.3 ± 2.17	0.04 ± 0.02
Cu:Zn	0.9 ± 0.2	2.12	18.52	17.8 ± 10.5	27.7 ± 23.6	14.9 ± 9.06	17.4 ± 11.5	43.6 ± 55.3

Note: All ratios are in μg/L:μg/L. Pb: lead. Fe: iron. Cu: copper. Zn: Zinc.

**Table 3:** Summary of inorganics in water samples for homes A and B during HBS and HBLD sampling.

Date	Pb Min	Pb Max	Pb Mean (±SD)	Pb RSD	Pb Median	PO4 Median	Fe Median	Cu Median	Zn Median
	μg/L	μg/L	μg/L	%	μg/L	mg/L	μg/L	μg/L	μg/L
**Home A**									
Apr 04, 2016	57.3	1180.0	346.2 (±254.8)	74	307.5	4.5	584.8	71.7	18.2
Apr 17, 2016	0.5	40.5	4.5 (±10.4)	229	1.5	3.7	40.4	3.6	6.6
May 05, 2016	18.6	212.7	72.3 (±49.2)	68	74.4	4.0	195.9	23.8	7.2
May 31, 2016	2.1	32.8	13.8 (±11.0)	80	11.0	3.8	77.5	8.7	6.4
Mar 09, 2017	0.5	279.9	25.9 (±73.32)	283	8.1	3.7	46.0	48.0	7.9
**Home B**									
Apr 04, 2016	14.5	775.9	239.1 (±204.1)	85	267.6	3.8	396.6	58.4	2.5
Apr 17, 2016	2.6	1308.0	251.2 (±408.4)	163	20.2	3.6	78.0	43.4	2.5
May 05, 2016	3.9	571.3	144.6 (±192.6)	133	50.6	3.5	92.7	32.6	2.5
May 31, 2016	17.1	632.3	166.4 (±173.2)	104	104.6	3.5	211.3	52.8	2.5
Mar 09, 2017	0.5	23.3	5.5 (±6.7)	122	2.5	3.6	91.0	87.7	2.5

Pb: lead. PO4: phosphate. Fe: iron. Cu: copper. Zn: zinc.

#### 3.2.2 Sources of lead in flushed water based on co-occurring metals and mapping the plumbing

The EPA mapped the water volume corresponding to the entire plumbing system, providing insights to the source of Pb in water flowing from the tap (Tables S1–S4). For home A and B, samples after the 10–12 L reflected water from the lead-free water mains (Fig. 1). However, this flushed water picked up Pb, resulting in levels up to 374.7 μg/L for home A and up to 1308 μg/L for home B, demonstrating the mobilization of Pb-sediment from the LSL or other sections of the plumbing during the sampling effort. The higher levels of Pb in high-velocity flushed samples indicates that low-velocity sampling is relatively ineffective in detecting the source of Pb in plumbing systems impacted by interruptions in CCT, if a large reservoir of Pb detached semi-randomly is a predominant source. Adding another dimension of sampling with higher flow rate without imposing any intervening stagnation event can unambiguously illustrate the nature of the problem, as scouring of deposits at high velocity will decisively reveal the presence of loose particulate Pb.

The co-occurrence of metals in the flushed water from the water main was that the strongest between Pb and phosphate in water over 2 rounds of sampling for home A and in 5 rounds of sampling for home B (Spearman’s ρ = 0.89 for home A, ρ = 0.77–0.92 for home B, Table S5). This relationship indicated that the previously formed passivated scale with incorporated orthophosphate in the LSL, detached into the water, which led to high levels of Pb correlated with high levels of phosphate. This finding highlights the importance of characterizing large reservoirs of loose deposits in the premise plumbing and that the risk of Pb release is not mitigated even after enhanced CCT.

In the flushed samples collected after 5 min of high rate flushing in both homes, a stable ratio of Pb:Cu of approximately 2:1 for home A and 5:1 for home B was always observed (Fig. S3), supporting a hypothesis that the dislodged Pb was somehow associated with Cu, which is most consistent with a deposition corrosion mechanism caused by the Cu pipe before the LSL at home B (Britton and Richards, 1981; Hu et al., 2012; St. Clair et al., 2012). Because orthophosphate might inhibit deposition corrosion, it is possible that these deposits formed in the more than 50 years these pipes were present before corrosion control was implemented in the latter part of the 20th century or in the year and a half period when orthophosphate was not added to the system in 2015–2016.

### 3.3 Impact of flow rate on Pb levels – comparing high-velocity and normal-velocity flushing

All samples collected from both home A and B using KTS sampling had median Pb < 5 μg/L both before and after LSL replacements, except for the first sampling at home A on Feb 02, 2016, which had a median Pb of 20.5 μg/L (Table S6). On Aug 16, 2016, at home B, the median Pb levels at the kitchen tap when sampled with the aerator on was 2.4 μg/L with a maximum spike of 7.9 μg/L. There was a marginal increase to median Pb of 3.1 μg/L and a maximum spike of 28.3 μg/L when the aerator was removed and the sampling was repeated immediately after the previous round with aerator on.

**Fig. 3:**
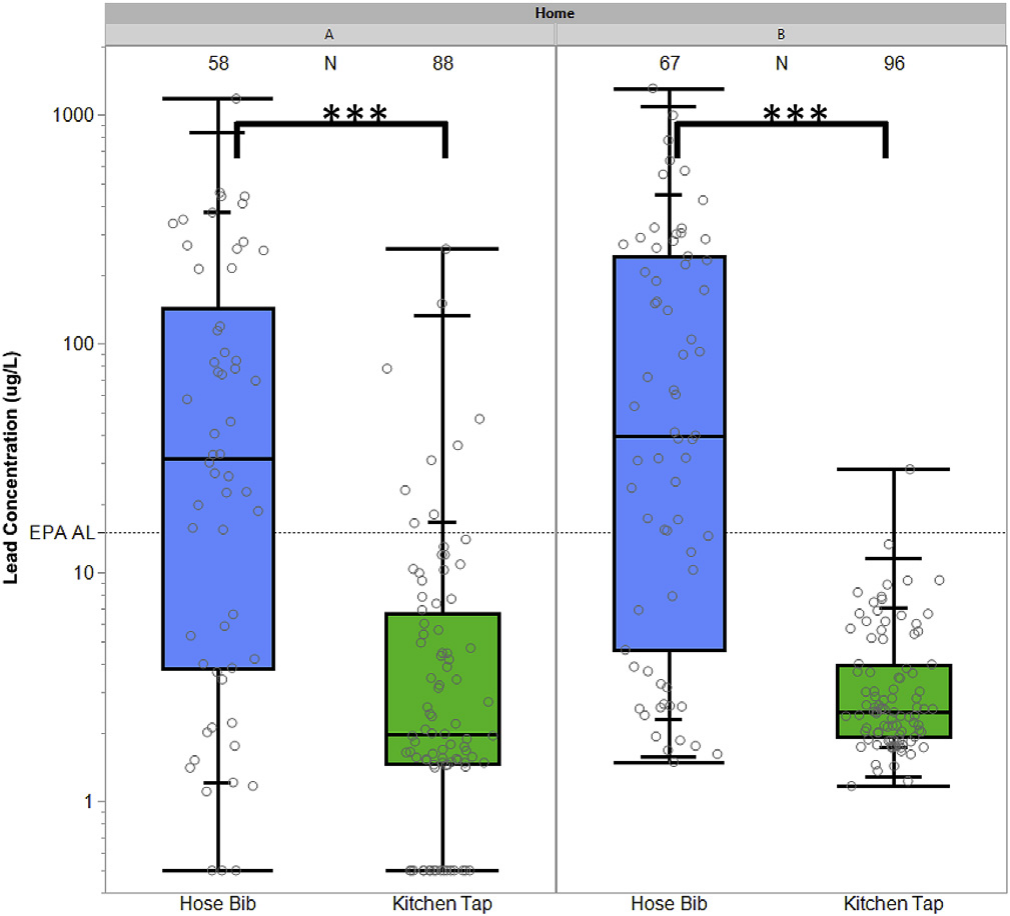
Lead concentrations in water as a function of sampling location for home A and B. Samples collected after LSL replacement (home A - Mar 09, 2017, and home B – Nov 01, 2016, Nov 08, 2016, and Mar 09, 2017) are not included. Hose bib samples were collected at high flow (12–13 L/min) and kitchen tap samples were collected at normal flow (2–3 L/min). Grey points represent the Pb concentrations. Boxes represent the interquartile range 25th-75th percentile. The dashed line represents the EPA Pb action level (AL) of 15 μg/L. Samples below the minimum reporting level (1.0 μg/L) were reported at 0.5 μg/L. Kruskal-Wallis test by ranks significance is denoted by (∗∗∗) implying p < 0.001.

The location of sampling within a home (either hose bib or kitchen tap), which is a surrogate for different sampling flow rates in this work, had a significant impact on Pb concentrations in water irrespective of sampling date (Kruskal-Wallis, p < 0.001) (Fig. 3). At home A, median Pb in water of 19.5 μg/L in samples collected from the hose bib at high flow (12–13 L/min) was over 10 times higher than median Pb in water of 1.8 μg/L in samples collected from kitchen tap at normal flow (2–3 L/min). Similarly, at home B, median Pb in water at high flow (12.3 μg/L) was over 5 times higher than the median Pb at normal flow (2.3 μg/L).

Sampling at low flow might not detect problems associated with a large reservoir of relatively loose Pb deposits from a service line, as was revealed by hose bib and high velocity sampling herein and in other studies (Cartier et al., 2012a; Clark et al., 2014; Pieper et al., 2017; Welter, 2016). Additionally, the current sampling procedure under the LCR requires the collection of a 1 L sample from the kitchen tap after at least 6-h of water stagnation, which likely underrepresents Pb problems from service lines (Clark et al., 2014; Del Toral et al., 2013; Pieper et al., 2017; Roy and Edwards, 2018).

**Fig. 4:**
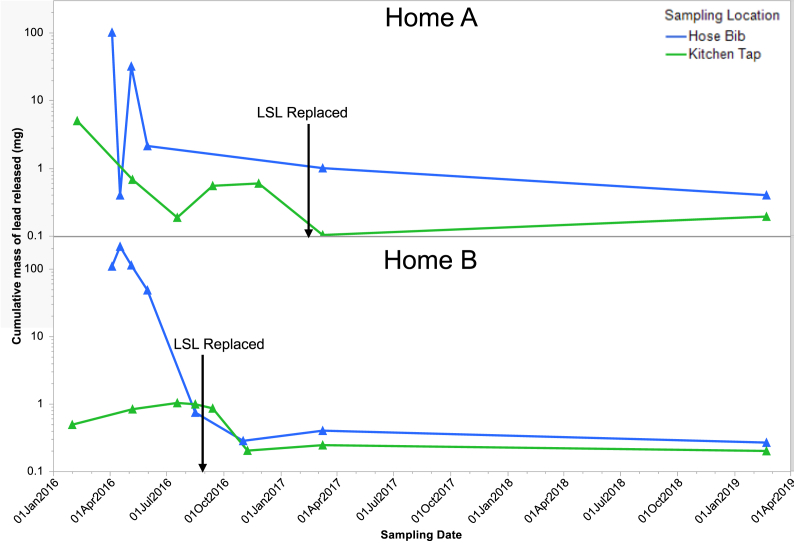
Calculated cumulative mass of Pb released as a function of sampling date normalized for 375 L of water flushed at home A and 405 L of water flushed at home B. Hose bib samples were collected at high flow (12–13 L/min) and kitchen tap samples were collected at normal flow (2–3 L/min).

For both homes, A and B, the cumulative mass of Pb released in each sampling round was calculated by integrating the area under the curve for Pb concentration versus volume flushed normalized to 375 L of water flushed in home A and 405 L at home B (Fig. 4). For hose bib sequential and kitchen tap sequential sampling rounds, the Pb concentration in the last sample was used for the rest of the volume of water to obtain a conservative estimate of cumulative mass of Pb released. At low velocity and after stagnation there was no significant difference in cumulative mass of Pb released from the hose bib when compared with kitchen tap in both homes (Mann-Whitney U test, p > 0.05). But the extra flushing at high flow from hose bib mobilized 23–100 times more Pb on average when compared to the samples collected at normal flow from kitchen tap. Moreover, in home B, the median cumulative mass of Pb released before LSL replacement from hose bib sampling was significantly greater than the cumulative mass released from kitchen tap (Mann-Whitney U test, p = 0.048). Nevertheless, in both homes, the cumulative mass of Pb released dropped below 1 mg per sampling round after LSL replacement, as the replacement removed the massive reservoir of Pb. Remedial flushing at high flow was effective in removing a higher mass of Pb deposits in the short term and may be an effective tool to remediate Pb contamination in premise plumbing and to track problems with loose deposits.

### 3.4 Implications

The City of Flint complied with the LCR in 2014–2015 using highly publicized loopholes (Roy and Edwards, 2018) such as pre-flushing the water and collecting water in narrow-mouth bottles at low flow (Davis et al., 2016; Del Toral, 2015; Pieper et al., 2017). On February 29, 2016, the EPA released a memorandum to provide clarifications for tap water sampling procedures and recommended using wide-mouth bottles and sampling at the highest possible normal flow rate (Davis et al., 2016; Pieper et al., 2017). This recommendation to use wide-mouth bottles has been incorporated into the proposed LCR revisions (EPA, 2019). Pb in drinking water is likely going to remain a significant concern for public health due to weak enforcement of the LCR, highly publicized loopholes in the LCR, rising concern about Pb in water exposures in the aftermath of the Flint water crisis, and increasing corrosivity of some source waters (Katner et al., 2018; Roy and Edwards, 2018).

## 4 Conclusions

In this study, we tracked the recovery of premise plumbing scale after water source switch and disruption of corrosion control in two problematic homes during the Flint water crisis. We employed remedial flushing at the highest possible flow at the hose bib to quantify the reservoir of particles in the plumbing and duration and magnitude of Pb release from that Pb reservoir during high flow throughout the recovery.

Elevated Pb levels measured in both homes from the hose bib at high flow showed that sampling from the kitchen tap alone would not have identified the true extent of Pb contamination. Although a downward trend in Pb levels was observed from January 2016–February 2019, a one-time high-velocity flow flush event was not effective in reducing Pb levels and might have even increased the risk of Pb release in the short term. By mapping Pb spikes with the plumbing profile and calculating metal ratios, we found that LSL was the major source for Pb, and LSL replacement was the most effective intervention for reducing Pb levels in the long term. Before LSL replacement, Pb levels in samples collected at high flow from the hose bib were significantly higher than samples collected at normal flow from the kitchen tap, irrespective of sampling date.

The long-term water quality problems in Flint arising from very expensive water, water main breaks, low water use, and disruption of scale due to abrupt change in water chemistry should have been anticipated. As water infrastructure ages and water conservation efforts are implemented nationwide, Pb in drinking water will likely remain a significant concern for the public and health agencies.

## Declaration of competing interest

The authors declare the following financial interests/personal relationships which may be considered as potential competing interests: Our data and testimony have been subpoenaed in several Flint water-related lawsuits. Dr. Marc Edwards has been subpoenaed as a fact witness in many of the lawsuits, but he has refused all financial compensation for time spent on those activities.
