# Supplementary material for: Tracking reduction of water lead levels in two homes during the Flint Federal Emergency
Source: Water Res X. 2020 Mar 3;7:100047. doi: 10.1016/j.wroa.2020.100047 (PMC7076093; doi:10.1016/j.wroa.2020.100047)
Supplement: Multimedia component [file mmc1.docx]

SUPPORTING INFORMATION

Tracking reduction of water lead levels in two homes during the Flint Federal Emergency

Anurag Mantha^a*^, Min Tang^a,b^, Kelsey J. Pieper^a,c^, Jeffrey L. Parks^a^, Marc A. Edwards^a^

**Affiliation and Address:**

^a^ Virginia Tech, Department of Civil and Environmental Engineering, 1145 Perry St., 418 Durham Hall, Blacksburg, Virginia 24061, United States

^b^ Current Affiliation: Oak Ridge Institute for Science and Education (ORISE) Postdoctoral Researcher at Environmental Protection Agency, 26 Martin Luther King Dr W., Cincinnati, OH 45220, United States (Email: [tang.min@epa.gov](mailto:tang.min@epa.gov); Phone: +1(540)750-6775)

^c^ Current Affiliation: Northeastern University, Civil and Environmental Engineering, Snell Engineering Center, Boston, MA 02115, United States

***Corresponding author:** Anurag Mantha, Phone: +1(540)750-6833, E-mail: anuragm@vt.edu, Address: 1145 Perry St., 418 Durham Hall, Blacksburg, Virginia, 24061, United States


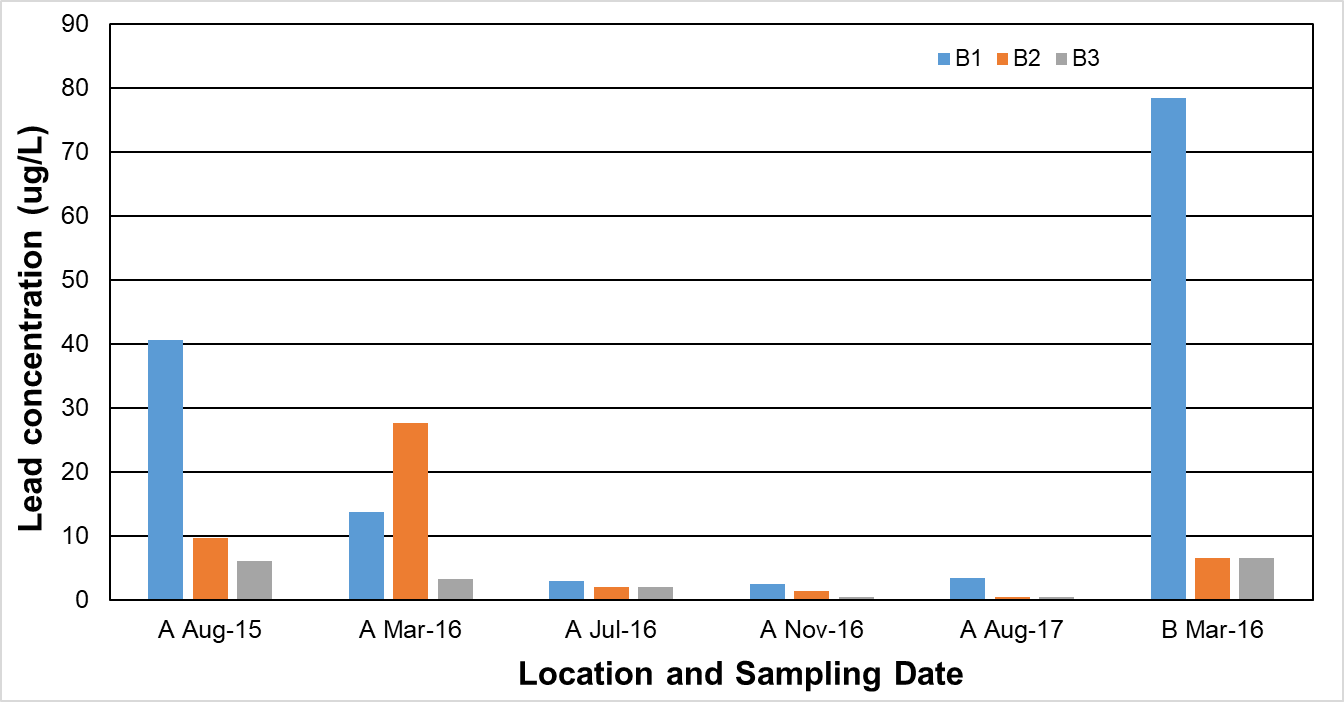


Figure S1. Lead levels at home A and B during five rounds of citizen sampling in Flint (Pieper et al. 2018). Home A was sampled in all five rounds, while home B was sampled only in March 2016. The lead sampling kits provided to residents had three bottles: bottle 1 (B1) was 1 L to be collected after 6-hours of stagnation, bottle 2 (B2) was 500 mL to be collected after 45 s of water flush at high flow after collecting B1, and bottle 3 (B3) was 125 mL to be collected after a subsequent 2 min of flushing at high flow.

**
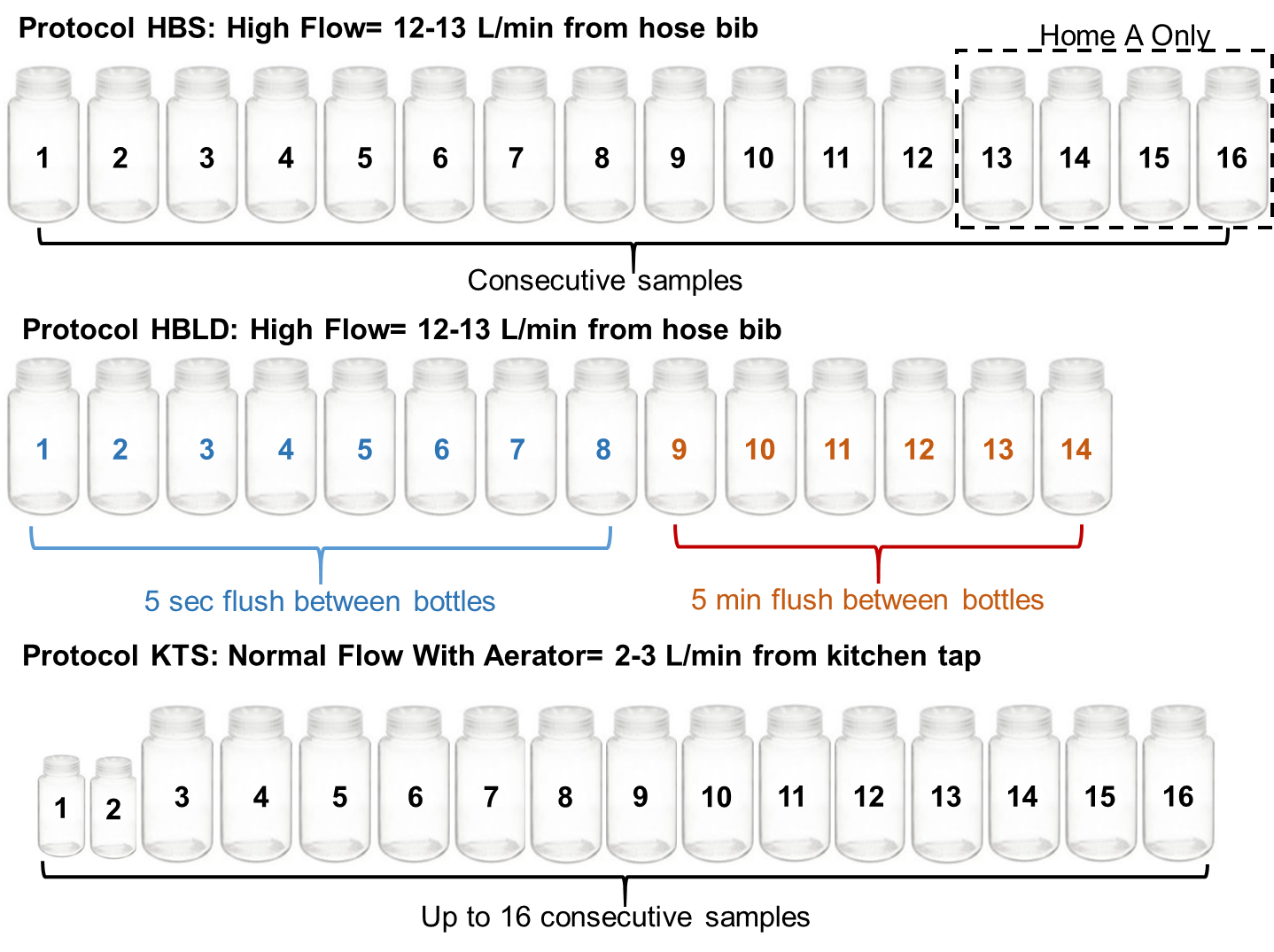
**

Figure S2. Sampling protocol for home A and B*.* Protocol HBS and HBLD samples were collected at high flow (12-13 L/min) from the hose bib and protocol KTS samples were collected at normal flow from the kitchen faucet. All bottles are of 1 L volume except bottles 1 and 2 for Protocol KTS (125 ml).


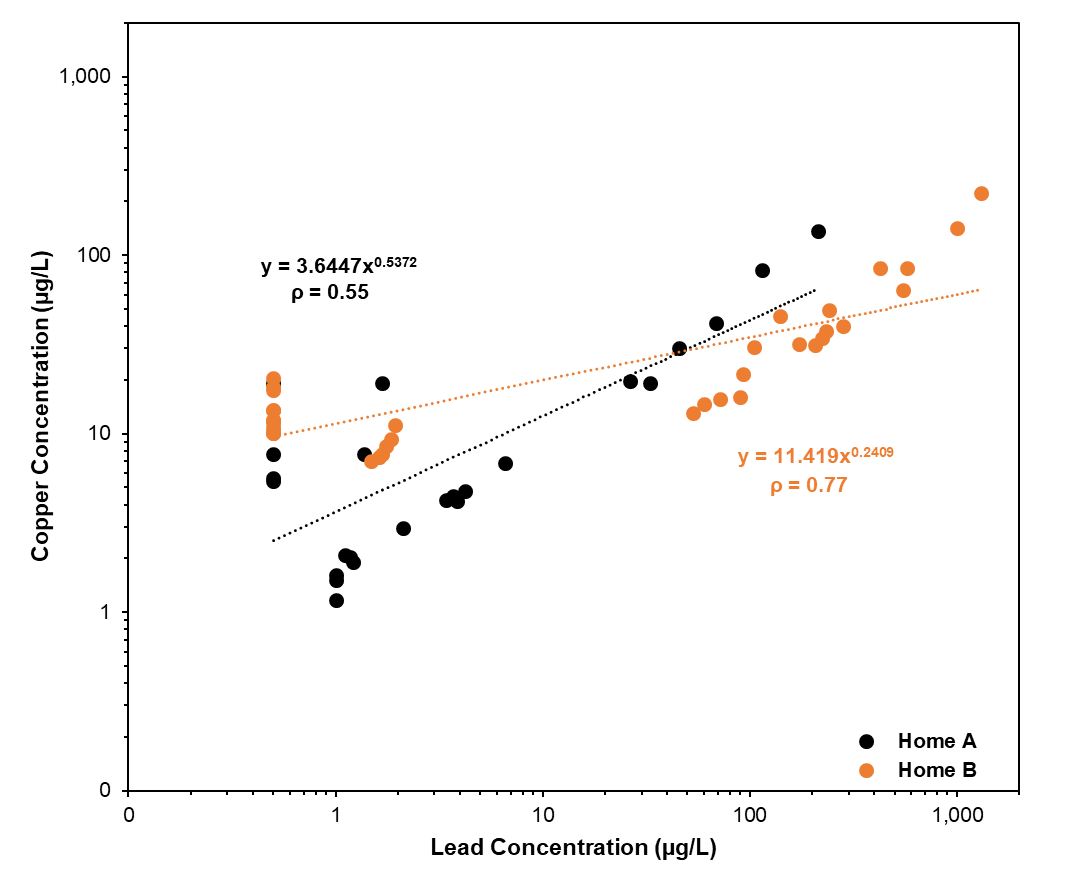


Figure S3. Lead vs. copper levels in well flushed samples collected in 5-minute intervals in both homes during hose bib long duration (HBLD) sampling. The number of samples (N) for home A = 24 and home B = 36.


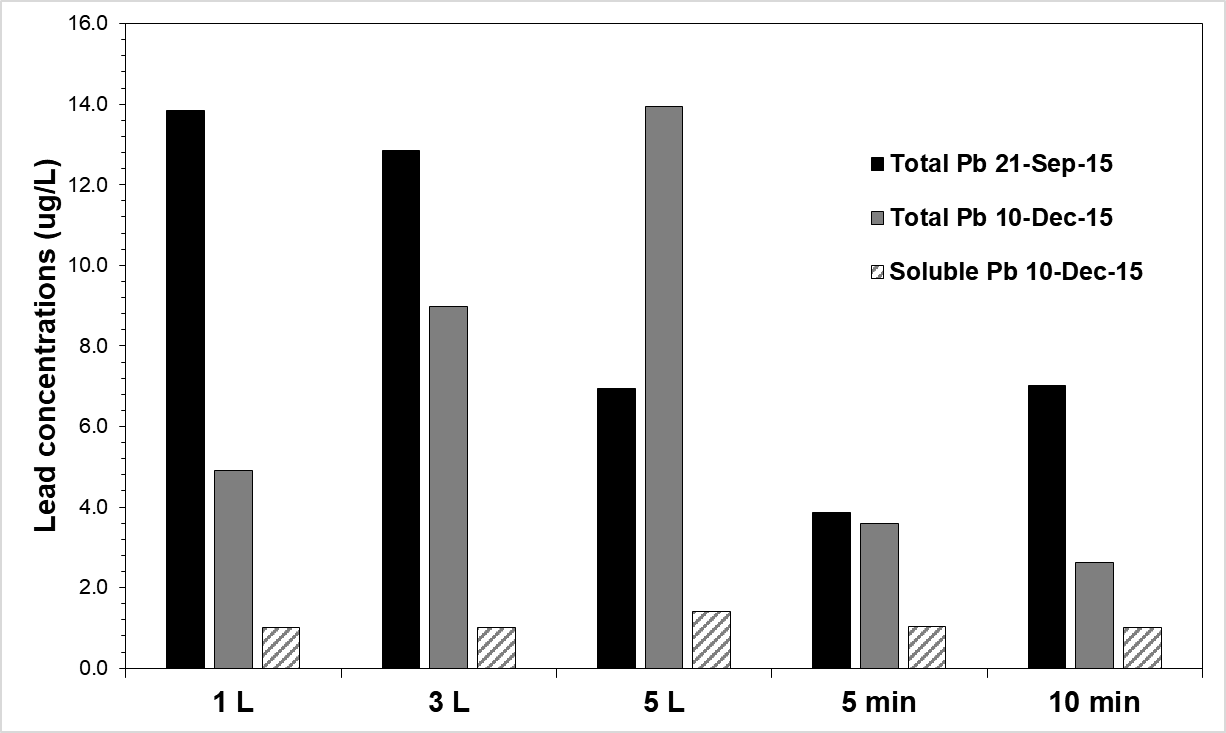


Figure S4. Total and soluble lead concentrations from a home in Flint, MI, which is not included in this study, during the period with the Flint River source (21 Sep, 2015) with no corrosion control treatment and after the switch back to the Lake Huron source and reintroduction of the enhanced corrosion control (10 Dec, 2015). All samples were collected in 1-L bottles after a 6-h stagnation for total lead and the samples were filtered thorough 0.45 µm filter to operationally determine the proportion of soluble lead.

Table S1. Home A plumbing survey from kitchen tap to service line conducted by EPA on March 24, 2016.

| No. | Item Description | Material | Length | Nominal ID | Volume | Volume | Cumulative Volume |
| --- | --- | --- | --- | --- | --- | --- | --- |
|  |  |  | **in.** | **in.** | **cu. in.** | **mL** | **L** |
| 1 | Pipe to Faucet | Copper | 6 | 0.375 | 0.7 | 10.9 | 0.01 |
| 2 | Fitting | Brass | 1 | 0.375 | 0.1 | 1.8 | 0.01 |
| 3 | Supply Tube | Braided Stainless Steel | 16 | 0.375 | 1.8 | 29.0 | 0.04 |
| 4 | Coupling | Brass | 1.5 | 0.375 | 0.2 | 2.7 | 0.04 |
| 5 | Pipe | Copper | 5.25 | 0.500 | 1.0 | 16.9 | 0.06 |
| 6 | Gate Valve | Brass | 1 | 0.500 | 0.2 | 3.2 | 0.06 |
| 7 | Pipe | Copper | 2 | 0.500 | 0.4 | 6.4 | 0.07 |
| 8 | Coupling | Copper | 0 | 0.500 | 0.0 | 0.0 | 0.07 |
| 9 | Pipe | Copper | 10 | 0.500 | 2.0 | 32.2 | 0.10 |
| 10 | Pipe | Copper | 6.75 | 0.500 | 1.3 | 21.7 | 0.12 |
| 11 | 90º fitting | Copper | 0.5 | 0.500 | 0.1 | 1.6 | 0.13 |
| 12 | Pipe | Copper | 3.75 | 0.500 | 0.7 | 12.1 | 0.14 |
| 13 | Coupling | Brass | 0 | 0.500 | 0.0 | 0.0 | 0.14 |
| 14 | Pipe | Copper | 13.25 | 0.500 | 2.6 | 42.6 | 0.18 |
| 15 | Street 45 | Copper | 0.5 | 0.500 | 0.1 | 1.6 | 0.18 |
| 16 | Tee | Copper | 0.5 | 0.500 | 0.1 | 1.6 | 0.18 |
| 17 | Pipe | Copper | 1.625 | 0.500 | 0.3 | 5.2 | 0.19 |
| 18 | Tee | Copper | 0.5 | 0.500 | 0.1 | 1.6 | 0.19 |
| 19 | Pipe | Copper | 11.5 | 0.500 | 2.3 | 37.0 | 0.23 |
| 20 | 90º fitting | Copper | 0.5 | 0.500 | 0.1 | 1.6 | 0.23 |
| 21 | Pipe | Copper | 16.75 | 0.500 | 3.3 | 53.9 | 0.28 |
| 22 | 90º fitting | Copper | 0.5 | 0.500 | 0.1 | 1.6 | 0.29 |
| 23 | Pipe | Copper | 2 | 0.500 | 0.4 | 6.4 | 0.29 |
| 24 | Tee | Copper | 0.5 | 0.500 | 0.1 | 1.6 | 0.29 |
| 25 | Pipe | Copper | 2.75 | 0.500 | 0.5 | 8.8 | 0.30 |
| 26 | Coupling | Copper | 0 | 0.500 | 0.0 | 0.0 | 0.30 |
| 27 | Pipe | Copper | 7 | 0.500 | 1.4 | 22.5 | 0.32 |
| 28 | Tee | Copper | 0.5 | 0.500 | 0.1 | 1.6 | 0.33 |
| 29 | Street 90 | Copper | 0.5 | 0.500 | 0.1 | 1.6 | 0.33 |
| 30 | Pipe | Copper | 67.5 | 0.500 | 13.3 | 217.2 | 0.55 |
| 31 | Tee | Brass | 0.5 | 0.500 | 0.1 | 1.6 | 0.55 |
| 32 | Pipe | Copper | 0.5 | 0.500 | 0.1 | 1.6 | 0.55 |
| 33 | Coupling | Copper | 0 | 0.500 | 0.0 | 0.0 | 0.55 |
| 34 | Pipe | Copper | 109 | 0.500 | 21.4 | 350.7 | 0.90 |
| 35 | 90º fitting | Copper | 0.5 | 0.500 | 0.1 | 1.6 | 0.90 |
| 36 | Pipe | Copper | 31.5 | 0.500 | 6.2 | 101.4 | 1.00 |
| 37 | Coupling | Copper | 0 | 0.500 | 0.0 | 0.0 | 1.00 |
| 38 | Pipe | Copper | 51.25 | 0.500 | 10.1 | 164.9 | 1.17 |
| 39 | 90º fitting | Copper | 0.5 | 0.500 | 0.1 | 1.6 | 1.17 |
| 40 | Pipe | Copper | 74.75 | 0.500 | 14.7 | 240.5 | 1.41 |
| 41 | 90º fitting | Copper | 0.5 | 0.500 | 0.1 | 1.6 | 1.41 |
| 42 | Pipe | Copper | 8.5 | 0.500 | 1.7 | 27.3 | 1.44 |
| 43 | 90º fitting | Brass | 0.5 | 0.500 | 0.1 | 1.6 | 1.44 |
| 44 | Nipple | Brass | 2.5 | 0.750 | 1.1 | 18.1 | 1.46 |
| 45 | Meter | Brass | 8 | 0.750 | 3.5 | 57.9 | 1.52 |
| 46 | Nipple | Brass | 2.5 | 0.750 | 1.1 | 18.1 | 1.53 |
| 47 | Street 90 | Galvanized | 0.75 | 0.750 | 0.3 | 5.4 | 1.54 |
| 48 | Gate Valve | Brass | 2.5 | 0.750 | 1.1 | 18.1 | 1.56 |
| 49 | Union (1x3/4) | Brass | 2.75 | 0.750 | 1.2 | 19.9 | 1.58 |
| 50 | Shut off | Lead | 2 | 1.000 | 1.6 | 25.7 | 1.60 |
| 51 | Service Line into Home | Lead | 432 | 1.000 | 339.3 | 5560.0 | 7.16 |

ID: inner diameter

Table S2. Home A plumbing survey from hose bib to service line conducted by Virginia Tech on August 8, 2017.

| No. | Item Description | Material | Length | Nominal ID | Volume | Volume | Cumulative Volume |
| --- | --- | --- | --- | --- | --- | --- | --- |
|  |  |  | **in.** | **in.** | **cu. in.** | **mL** | **L** |
| 1 | Pipe | Copper | 11 | 0.750 | 4.9 | 79.6 | 0.08 |
| 2 | Pipe | Copper | 5 | 0.750 | 2.2 | 36.2 | 0.12 |
| 3 | Pipe | Copper | 141 | 0.750 | 62.3 | 1020.8 | 1.14 |
| 4 | Pipe | Copper | 70 | 0.750 | 30.9 | 506.8 | 1.64 |
| 5 | Pipe | Copper | 144 | 0.750 | 63.6 | 1042.5 | 2.69 |
| 6 | Pipe | Copper | 84 | 0.750 | 37.1 | 608.1 | 3.29 |
| 7 | Pipe | Copper | 70 | 0.750 | 30.9 | 506.8 | 3.80 |
| 8 | Pipe | PEX | 4 | 0.5 | 0.8 | 12.9 | 3.81 |
| 9 | Pipe | PEX | 21.5 | 0.75 | 9.5 | 155.7 | 3.97 |
| 10 | Meter | Brass | 8 | 0.750 | 3.5 | 57.9 | 4.03 |
| 11 | Nipple | Brass | 2.5 | 0.750 | 1.1 | 18.1 | 4.05 |
| 12 | Street 90 | Galvanized | 0.75 | 0.750 | 0.3 | 5.4 | 4.05 |
| 13 | Gate Valve | Brass | 2.5 | 0.750 | 1.1 | 18.1 | 4.07 |
| 14 | Union (1x3/4) | Brass | 2.75 | 0.750 | 1.2 | 19.9 | 4.09 |
| 15 | Shut off | Lead | 2 | 1.000 | 1.6 | 25.7 | 4.11 |
| 16 | Service Line into Home | Lead | 432 | 1.000 | 339.3 | 5560.0 | 9.67 |

ID: inner diameter

Table S3. Home B plumbing survey from kitchen tap to service line conducted by EPA on March 21, 2016.

| No. | Item Description | Material | Length | Nominal ID | Volume | Volume | Cumulative Volume |
| --- | --- | --- | --- | --- | --- | --- | --- |
|  |  |  | **in.** | **in.** | **cu. in.** | **mL** | **L** |
| 1 | Pipe | Copper | 3 | 0.375 | 0.3 | 5.4 | 0.01 |
| 2 | Nut | Brass | 1 | 0.375 | 0.1 | 1.8 | 0.01 |
| 3 | Pipe | Plastic | 1 | 0.375 | 0.1 | 1.8 | 0.01 |
| 4 | Compression coupling | Brass | 1 | 0.375 | 0.1 | 1.8 | 0.01 |
| 5 | Pipe | Copper | 1 | 0.375 | 0.1 | 1.8 | 0.01 |
| 6 | Copper fitting/reducer | Copper | 1 | 0.375 | 0.1 | 1.8 | 0.01 |
| 7 | Pipe | Copper | 5 | 0.500 | 1.0 | 16.1 | 0.03 |
| 8 | Gate valve | Brass | 2 | 0.500 | 0.4 | 6.4 | 0.04 |
| 9 | Pipe | Copper | 4 | 0.500 | 0.8 | 12.9 | 0.05 |
| 10 | 90º Elbow | Copper | 1 | 0.500 | 0.2 | 3.2 | 0.05 |
| 11 | Pipe | Copper | 4 | 0.500 | 0.8 | 12.9 | 0.07 |
| 12 | 90º Elbow | Copper | 1 | 0.500 | 0.2 | 3.2 | 0.07 |
| 13 | Pipe | Copper | 26 | 0.500 | 5.1 | 83.7 | 0.15 |
| 14 | 90º Elbow | Copper | 1 | 0.500 | 0.2 | 3.2 | 0.16 |
| 15 | Pipe | Copper | 12 | 0.500 | 2.4 | 38.6 | 0.19 |
| 16 | 90º Elbow | Copper | 1 | 0.500 | 0.2 | 3.2 | 0.20 |
| 17 | Pipe | Copper | 16 | 0.500 | 3.1 | 51.5 | 0.25 |
| 18 | Gate valve | Brass | 2.5 | 0.500 | 0.5 | 8.0 | 0.26 |
| 19 | Pipe | Copper | 2.5 | 0.500 | 0.5 | 8.0 | 0.27 |
| 20 | Tee (Reducing) | Copper | 1 | 0.750 | 0.4 | 7.2 | 0.27 |
| 21 | Pipe | Copper | 54.5 | 0.500 | 10.7 | 175.4 | 0.45 |
| 22 | Tee (Reducing) | Copper | 1 | 0.750 | 0.4 | 7.2 | 0.46 |
| 23 | Pipe | Copper | 2 | 0.750 | 0.9 | 14.5 | 0.47 |
| 24 | 90º Elbow | Copper | 1 | 0.750 | 0.4 | 7.2 | 0.48 |
| 25 | Pipe | Copper | 1 | 0.750 | 0.4 | 7.2 | 0.48 |
| 26 | 90º Elbow | Copper | 1 | 0.750 | 0.4 | 7.2 | 0.49 |
| 27 | Pipe | Copper | 4 | 0.750 | 1.8 | 29.0 | 0.52 |
| 28 | 90º Elbow | Copper | 1 | 0.750 | 0.4 | 7.2 | 0.53 |
| 29 | Pipe | Copper | 4 | 0.750 | 1.8 | 29.0 | 0.56 |
| 30 | 90º Elbow | Copper | 1 | 0.750 | 0.4 | 7.2 | 0.56 |
| 31 | Pipe | Copper | 4 | 0.750 | 1.8 | 29.0 | 0.59 |
| 32 | Tee | Copper | 1 | 0.750 | 0.4 | 7.2 | 0.60 |
| 33 | Street 90 | Copper | 1 | 0.750 | 0.4 | 7.2 | 0.61 |
| 34 | Pipe | Copper | 5 | 0.750 | 2.2 | 36.2 | 0.64 |
| 35 | 90º Elbow | Copper | 1 | 0.750 | 0.4 | 7.2 | 0.65 |
| 36 | Pipe | Copper | 127 | 0.750 | 56.1 | 919.4 | 1.57 |
| 37 | 90º Elbow | Copper | 1 | 0.750 | 0.4 | 7.2 | 1.58 |
| 38 | Pipe | Copper | 33 | 0.750 | 14.6 | 238.9 | 1.82 |
| 39 | 90º Elbow | Copper | 1 | 0.750 | 0.4 | 7.2 | 1.82 |
| 40 | Pipe | Copper | 32.5 | 0.750 | 14.4 | 235.3 | 2.06 |
| 41 | Gate Valve | Brass | 2 | 0.750 | 0.9 | 14.5 | 2.07 |
| 42 | Pipe | Copper | 15.5 | 0.750 | 6.8 | 112.2 | 2.19 |
| 43 | 90º Elbow | Copper | 1 | 0.750 | 0.4 | 7.2 | 2.19 |
| 44 | Fitting (Pipe) | Copper | 2 | 0.750 | 0.9 | 14.5 | 2.21 |
| 45 | Street 90 | Copper | 1 | 0.750 | 0.4 | 7.2 | 2.21 |
| 46 | Female adapter | Copper | 1 | 0.750 | 0.4 | 7.2 | 2.22 |
| 47 | Nipple | Brass | 2 | 0.750 | 0.9 | 14.5 | 2.24 |
| 48 | Meter | Brass | 6 | 0.750 | 2.7 | 43.4 | 2.28 |
| 49 | 90º union shutoff | Brass | 2 | 0.750 | 0.9 | 14.5 | 2.29 |
| 50 | Service Line into Home | Copper | 480 | 0.750 | 212.1 | 3475.0 | 5.77 |
| 51 | City Portion Service Line | Lead | 480 | 1.000 | 377.0 | 6177.8 | 11.95 |

ID: inner diameter

Table S4. Home B plumbing survey from hose bib to service line conducted by Virginia Tech on August 8, 2017.

| No. | Item Description | Material | Length | Nominal ID | Volume | Volume | Cumulative Volume |
| --- | --- | --- | --- | --- | --- | --- | --- |
|  |  |  | **in.** | **in.** | **cu. in.** | **mL** | **L** |
| 1 | Pipe | Copper | 10 | 0.750 | 4.4 | 72.4 | 0.07 |
| 2 | Pipe | Copper | 2 | 0.750 | 0.9 | 14.5 | 0.09 |
| 3 | Pipe | Copper | 2 | 0.750 | 0.9 | 14.5 | 0.10 |
| 4 | Pipe | Copper | 5 | 0.750 | 2.2 | 36.2 | 0.14 |
| 5 | Pipe | Copper | 5 | 0.750 | 2.2 | 36.2 | 0.17 |
| 6 | Pipe | Copper | 5 | 0.750 | 2.2 | 36.2 | 0.21 |
| 7 | Pipe | Copper | 2 | 0.750 | 0.9 | 14.5 | 0.22 |
| 8 | Pipe | Copper | 7 | 0.750 | 3.1 | 50.7 | 0.28 |
| 9 | Pipe | Copper | 124 | 0.750 | 54.8 | 897.7 | 1.17 |
| 10 | Pipe | Copper | 32 | 0.750 | 14.1 | 231.7 | 1.40 |
| 11 | Pipe | Copper | 51 | 0.750 | 22.5 | 369.2 | 1.77 |
| 12 | Pipe | Copper | 4 | 0.750 | 1.8 | 29.0 | 1.80 |
| 13 | Pipe | Copper | 5 | 0.750 | 2.2 | 36.2 | 1.84 |
| 14 | meter | Brass | 6 | 0.750 | 2.7 | 43.4 | 1.88 |
| 15 | 90^o^ union shutoff | Brass | 2 | 0.750 | 0.9 | 14.5 | 1.90 |
| 16 | Service Line into Home | Copper | 480 | 0.750 | 212.1 | 3475.0 | 5.37 |
| 17 | City Portion Service Line | Lead | 480 | 1.000 | 377.0 | 6177.8 | 11.55 |

ID: inner diameter

Table S5. Spearman's rank correlation coefficients (ρ) and mean ratios for metals at home A and B during HBS and HBLD sampling

| **Dates** | **Pb:Fe** | | **Pb:PO4** | | **Pb:Zn** | | **Cu:Zn** | |
| --- | --- | --- | --- | --- | --- | --- | --- | --- |
|  | **ρ** | **µg/L: µg/L** | **ρ** | **µg/L: mg/L** | **ρ** | **µg/L: µg/L** | **ρ** | **µg/L: µg/L** |
| **HOME A** | | | | | | | | |
| **Apr 4, 2016** | 0.90 | 0.50 ± 0.14 | 0.89 | 74.36 ± 50.02 | -0.10^§^ | 17.26 ± 11.62 | 0.93 | 3.77 ± 1.18 |
| **Apr 17, 2016** | 0.15^§^ | 0.08 ± 0.14 | -0.65 | 1.78 ± 4.85 | 0.95 | 0.28 ± 0.13 | 0.95 | 0.63 ± 0.13 |
| **May 5, 2016** | 0.73 | 0.40 ± 0.13 | 0.89 | 17.74 ± 10.27 | -0.13^§^ | 16.60 ± 21.1 | 0.13^§^ | 9.49 ± 14.07 |
| **May 31, 2016** | 0.38^§^ | 0.17 ± 0.14 | -0.45^§^ | 3.80 ± 3.04 | 0.69 | 2.10 ± 1.12 | 0.90 | 1.71 ± 0.67 |
| **Mar 9, 2017** | 0.94 | 0.24 ± 0.24 | 0.43^§^ | 8.04 ± 23.8 | 0.93 | 0.68 ± 0.42 | 0.84 | 9.06 ± 7.86 |
| **HOME B** | | | | | | | | |
| **Apr 4, 2016** | 0.90 | 0.56 ± 0.20 | 0.81 | 62.04 ± 49.95 | -0.53^§^ | 85.47 ± 86.7 | 0.68 | 17.79 ± 10.50 |
| **Apr 17, 2016** | 0.97 | 0.64 ± 0.54 | 0.82 | 60.51 ± 91.93 | -0.60 | 99.97 ± 163.7 | 0.40^§^ | 27.64 ± 23.59 |
| **May 5, 2016** | 0.85 | 0.77 ± 0.52 | 0.89 | 38.96 ± 50.21 | -0.77 | 56.71 ± 77.83 | 0.51^§^ | 14.89 ± 9.06 |
| **May 31, 2016** | 0.66 | 0.45 ± 0.26 | 0.92 | 38.18 ± 30.96 | -0.22 | 49.19 ± 41.85 | 0.90 | 17.40 ± 11.46 |
| **Aug 16, 2016** | -0.16^§^ | 0.43 ± 0.17 | 0.35^§^ | 0.86 ± 0.44 | 0.87 | 0.77 ± 0.37 | 0.96 | 4.95 ± 2.34 |
| **Nov 1, 2016** | 0.16^§^ | 0.09 ± 0.12 | -0.13^§^ | 0.59 ± 0.55 | 0.88 | 0.21 ± 0.10 | 0.80 | 12.59 ± 19.77 |
| **Mar 9, 2017** | 0.81 | 0.03 ± 0.03 | 0.77 | 1.33 ± 1.48 | 0.93 | 1.37 ± 1.45 | 0.93 | 43.62 ± 55.33 |

^§^Spearman’s rank correlation p > 0.05

Pb: lead. PO4: phosphate. Fe: iron. Cu: copper. Zn: zinc.

Table S6. Summary of lead and copper levels at home A and B during KTS sampling

| **Dates** | **Pb Min** | **Pb Max** | **Median Pb** | **Median Cu** |
| --- | --- | --- | --- | --- |
|  | **µg/L** | **µg/L** | **µg/L** | **µg/L** |
| **HOME A** | | | | |
| **Feb 2, 2016** | 6.0 | 260.0 | 20.5 | 40.0 |
| **May 7, 2016** | 1.6 | 10.9 | 2.4 | 14.6 |
| **Jul 18, 2016** | 0.3 | 16.5 | 0.3 | 135.0 |
| **Sep 13, 2016** | 1.4 | 7.9 | 1.6 | 6.4 |
| **Nov 26, 2016** | 1.5 | 10.4 | 1.8 | 5.8 |
| **Feb 14, 2016** | LSL replaced | | | |
| **Mar 9, 2017**^§⁑^ | 0.3 | 8.3 | 0.3 | 17.4 |
| **Feb 21, 2019**^§⁑^ | 0.3 | 3.5 | 0.7 | 10.0 |
| **HOME B** | | | | |
| **Jan 31, 2016** | 1.2 | 7.4 | 1.9 | 80.0 |
| **May 7, 2016** | 1.8 | 9.3 | 2.3 | 122.0 |
| **Jul 18, 2016** | 2.0 | 8.9 | 2.5 | 78.9 |
| **Aug 16, 2016**^⁑^ | 1.7 | 7.9 | 2.4 | 57.1 |
| **Aug 16, 2016**^§⁑^ | 2.4 | 28.3 | 3.1 | 25.5 |
| **Sep 13, 2016** | 1.7 | 7.7 | 2.1 | 69.9 |
| **Sep 28, 2016** | LSL replaced | | | |
| **Nov 8, 2016** | 0.3 | 5.2 | 0.7 | 155.0 |
| **Mar 9, 2017**^§⁑^ | 0.3 | 37.8 | 1.1 | 223.1 |
| **Feb 21, 2019**^§⁑^ | 0.3 | 0.3 | 0.3 | 34.9 |

^§^KTS sampling conducted with the aerator removed.

^⁑^KTS sampling conducted by Virginia Tech using EPA Protocol

KTS: kitchen tap sequential sampling.

Pb: lead. Cu: copper.
